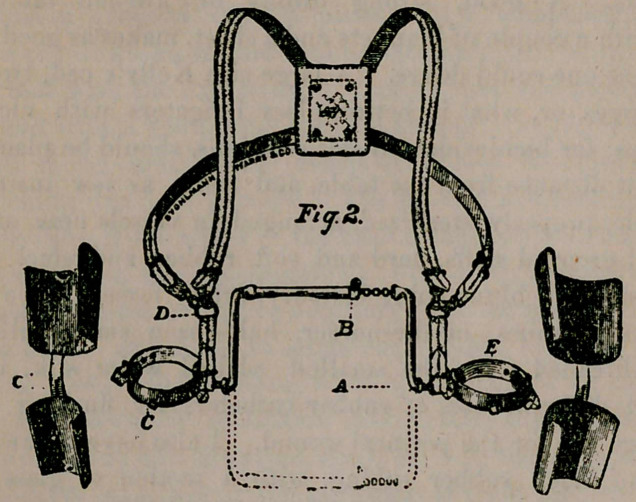# Remarks on the Technique of External Urethrotomy

**Published:** 1896-09

**Authors:** W. S. Goldsmith

**Affiliations:** Atlanta, Ga.; Lecturer on Genito-Urinary and Rectal Diseases, Atlanta Medical College


### REMARKS ON THE TECHNIQUE OF EXTERNAL URETHROTOMY.

By W. S. GOLDSMITH, M.D., Atlanta, Ga.

Lecturer on Genito-Urinary and Rectal Diseases, Atlanta Medical College.

To my mind there is not in the whole range of surgery an operation, if intelligently executed, fraught with better results and comfort to the patient, than an external urethrotomy. At the same time, it is an operation that is regarded as dangerous, difficult, and, I regret to say, advised as unnecessary by men who are themselves capable of doing good work in this line. For this reason I am constrained to contribute a few suggestions which I am sure are sound, plain, and practicable. It is not within the province of this paper to discuss the various varieties of stricture, their etiology or pathology, and I shall confine myself to the elucidation of the different steps of the operation, together with brief references to the appliances necessary to its successful accomplishment.

I have seen within the past few years a number of cases illustrating the most careless and barbarous use of instruments, during a course of treatment for the correction of some constriction in the urethra. This fact leads me to assert that no man is justified in doing this operation, or any other for that matter, in the genito-urinary tract, who does not practice the most extreme delicacy of touch and gentleness in the manipulation of any urethral instrument, be it either the softest rubber catheter or largest steel sound. The physician guilty of such indiscretion, and I rather suspect there are quite a number in the profession, should not dare to jeopardize the life of a man by ignorantly and blindly "rushing in where angels fear to tread." We are told by eminent surgeons and gynecologists of the fearful slaughter of the innocents perpetrated by the mushroom abdominal surgeon and pseudo-gynecologist, but not one note of warning has emanated advising us of the bad results at the hands of tinkering stricture doctors.

What surgeon has not heard the oft-repeated story of the patient having an impermeable stricture, or one with false passages, ascribe his condition to the physician whom he first consulted. The days of rough-and-ready grand-stand surgery are numbered in the past, and to-day the physician who is lacking in the many little refinements of manipulation is relegated to the class to which he belongs.

The inability to introduce a guide is one of the principal objections urged against this operation, and we all agree that it is a matter of much tediousness to locate the urethra without this valuable aid. With a grooved staff in place, it is a comparatively easy matter to cut down on it, and rapidly complete our work. But I contend that with the expenditure of more time and nicety of dissection, and with a good, long incision through the bulb, down on the point of the sound, little difficulty should be experienced in accomplishing the end in view. The profuse hemorrhage following the division of the artery of the bulb is the objection to a long anterior incision, but this can largely be obviated by carefully ligating such bleeding points as may be necessary to procure an unobstructed operating area. If we fail to introduce a guide, even one or more filiforms in a well oiled urethra, when the patient is in the prone position and muscular relaxation is complete, it is useless to continue our efforts in this direction when he is in the lithotomy position, and every structure of the perineum is tense and unyielding, the tract of the urethra changed, and other familiar landmarks obliterated. This brings to mind an operation I witnessed some time ago, at which three surgeons of some note overlooked the necessity of introducing a guide until the patient was in position for operation. I was astonished at the persistency with which all manner of hard and soft instruments were pushed and punched in that man's urethra without success. Such faulty technique was absolutely uncalled for, and was productive of free hemorrhage and undoubted contusion and laceration of the urethral wall.

The preparation of the patient varies according to the length of time he has been affected, and the condition of the bladder and kidneys. A thorough examination of the urine is made, and any abnormal condition corrected as far as possible. In strictures where the lumen is so small as to preclude a steady stream, not much can be done, except in a general way, until thorough drainage is effected subsequent to the operation. As a routine practice, however, I administer quinine and salol, five grains each, every six hours, for twenty-four or thirty-six hours preceding operation, and in conjunction advise the use of a good lithia water, preferably the Bowden Lithia, on account of its palatability and the ease with which patients imbibe a quantity, without feeling any untoward effect. The usual salines are given the night before, and all hair is shaved from the pubis, perineum, nates, and half way down the thighs. A good, strong dining or kitchen table, well padded with a couple of blankets and a sheet, makes as good operating table as one could desire. A large size Kelly's pad, two fountain syringes, or, what is better, glass irrigators with nicely adjusted caps, for bichloride and salt solutions, should be placed at a convenient distance from the table, and lastly as few instruments as possible, properly sterilized, arranged in vessels near at hand. The usual grooved staffs, hard and soft rubber and steel sounds, scalpels, straight, blunt-point bistoury, artery forceps, grooved director, steel probes, needle-holder, half-dozen small half-curved needles threaded with the smallest plaited white silk, urethretome, and different sizes of rubber catheters for flushing urethra and a large one for the perineal wound. I also have at hand five or six feet of black rubber tubing, with a section of glass tubing fitted in each end, one of which connects with the perineal tube, while the other projects through a clean cork into a two-quart white glass bottle, which is the receptacle for the urine. This bottle is kept out of the way under the bed and its contents measured once in twenty-four hours, and a memoranda of the same kept during the period of convalescence. Two assistants are necessary, exclusive of the administrator of the anesthetic, while a third could be utilized to advantage. The two supporting the legs of the patient should have their duties clearly outlined, the one on the operator's right being familiar with the use of all the urethral instruments, and fully appreciating the importance of the absolutely correct position the staff should assume, while the other sponges and looks after the knives, forceps, needles, and ligatures. The accuracy

Fig 1.

Fig. 2.

and steadiness with which the patient is maintained in position is of prime importance, and this precaution should be insisted on until the wound is finally dressed. The very best apparatus for this purpose is a modification of Clover's crutch, so ingeniously made by Dr. F. Tilden Brown, Presbyterian Hospital, New York. I consider this part of the technique so important, and this crutch such an excellent one, that I take the liberty of reproducing herewith, at length, Dr. Brown's description of this admirable appliance, together with cuts, for the use of which I thank Messrs. Tieman & Co., New York.

" . . . . The main faults in the Clover crutch are the straight bar and neck-strap. The former seriously invades the operative territory. In urethrotomy it is near enough to the penis to encroach upon some part of the arc which the handle of a sound or grooved staff must traverse to enter or leave the urethra. It invariably causes the operator to use undue force and a false lateral position to crowd the handle of the instrument under the bar. In operations upon the rectum, perineum, or vagina this straight bar hung with sterilized cloths shuts off light from the field of operation, and fills the space convenient for instruments. The single neck-strap is faulty in that it exerts a considerable pressure upon the vessels and nerves of the neck, the deleterious effects of which are clearly marked in the congested state of the head, and the unsatisfactory respiration during anesthesia. These phenomena are particularly noticeable when the patient is flabby, plethoric, or alcoholic—that is, intensifies the dangers incidental to the anesthetic state commonly noticed in just such cases. To obviate these objections to the old apparatus, an extension bar, with right-angled elbows, continued into parallel arms, six inches long, has been substituted. These arms swivel in the collar of the metal leg-crutch. By this means the extension-bar can be turned well out of the way. It is ordinarily kept flat upon the patient's abdomen. The former thumb screw on the extension bar has been replaced by a spring catch, which, in spreading the thighs to the desired degree, is automatic, and maintains the position, the spring needing only to be pressed upon when the thighs are again to be abducted. Instead of a single neck-strap with two buckles as used in the original Clover, counter extension is effected by both neck and side-straps, radiating from a back pad. When the thighs are once flexed, all of the traction should be maintained by the two side straps, no pressure at all being exerted upon the supraclavicular region by the neck-strap.

" . . . . The drooping downward and inward of the feet is also troublesome; for while the knees are bent, the more the thighs are abducted, the greater the approximation of the feet. In the hospital amphitheater, where it is particularly desirable to afford a large visual angle of the operative field, this encroachment of the feet and lower legs is quite annoying. To avoid this extreme knee flexion must be prevented, and the leg rests, which button on to the leg-crutches, are introduced to obviate it. These leg-rests are made of light metal, coupled by a slightly-angled strong steel brace. These permit the natural knee flexion necessary when the thighs are also flexed."

In the absence of a crutch I have secured fixation of the legs in flexed position by firmly tying the hands and ankles of the patient, but it requires the constant attention of both assistants to hold the legs abducted to a degree necessary to keep the anatomical landmarks in a position by which there will occur no deviation of the urethral tract from the median line. Again, the use of this crude arrangement curtails the field of operation to such an extent as to obstruct the light, and render uncomfortable and awkward the movements of the operator. The original incision should be below the bulbous portion of the urethra, and in ordinary cases it will, with careful dissection, suffice. If, however, this particular surface of the perineum is short, and the urethra pushed backwards and deflects from the normal course, we are then justified in extending the incision upward through the bulb. The most difficult part of this operation, when done without a guide, is distinguishing the urethra from the surrounding structures. When it is located, and here great care must be exercised, we may cut directly down on stricture tissue. A steel probe or hard rubber sound of small size should be frequently used, both anteriorly and posteriorly, in the endeavor to locate the free opening of the urethra, and in a moderately dry wound the mucous membrane can easily be distinguished by its gray and shining appearance. When this is found, and after the constrictions have been severed, I carefully dilate the posterior portion of the canal with large sounds, and try to introduce my finger into the bladder. There are nearly always one or more bands anterior to the perineal wound, which are incised with the urethretome to a size sufficient to admit the sound I think is indicated in the after-treatment. In my experience the cicatrix following an extensive incision in the urethra rapidly disappears when the functions of the canal are restored and proper instrumentation inaugurated. The canal is irrigated with a warm 1 to 5000 bichloride solution and cleared of all blood-clots, and the bladder thoroughly washed out with warm salt solution. The perineal tube for bladder drainage is of large size and has three or four holes in the distal end, thus insuring perfect drainage and obviating the otherwise frequent necessity of withdrawing it to be cleansed. This tube is fastened in place by a smooth, sterilized, nickel-plated safety-pin, which catches up the skin at the margin of the incision, and barely pierces the lumen of the tube. A ligature used for the same purpose should provoke more objections than this method. Other appliances are never reliable, since their intricate mechanical construction is such as to invite sepsis, discomfort to the patient, and aggravation to the medical attendant. Narrow strips of iodoform gauze are packed in and around the wound, then bichloride gauze and cotton, and over all a T bandage applied, through which the tube finds its exit. The bladder and urethra should be irrigated the next and each succeeding day for a week with warm 1 to 6000 permanganate of potash solution. I do not introduce instruments into the penile portion of the urethra, except through the meatus, in the event of its section, until the drainage-tube is removed. This latter can be withdrawn, usually on the fifth or sixth day, if favorable progress warrants. For lubricating purposes I use sterilized sweet oil at the time of operation and subsequently, and if the urethra is very sensitive, and much pain experienced during instrumentation, two per cent. cocaine is added to the oil. I think it unwise to introduce the sound to the deep urethra oftener than every four days, and then the bladder should be approached by degrees carrying the instrument deeper and deeper at each séance.

27–28 *Grant Building.*

---

### GUAIACOL FOR TUBERCULOUS IRITIS.

Dr. Vignes (*Bulletin Méd.*, April 22, 1896,) had under his care a case of tuberculous iritis, which the usual treatment did not relieve. The treatment consisted of administration of mydriatics, paracentesis, hydrargyrum, salicylates, revulsion, etc. He then had recourse to oily injections (1:15) of guaiacol, and the result was remarkable; the most rapid improvement following.

## Figures and Tables

**Fig. 1. f1:**
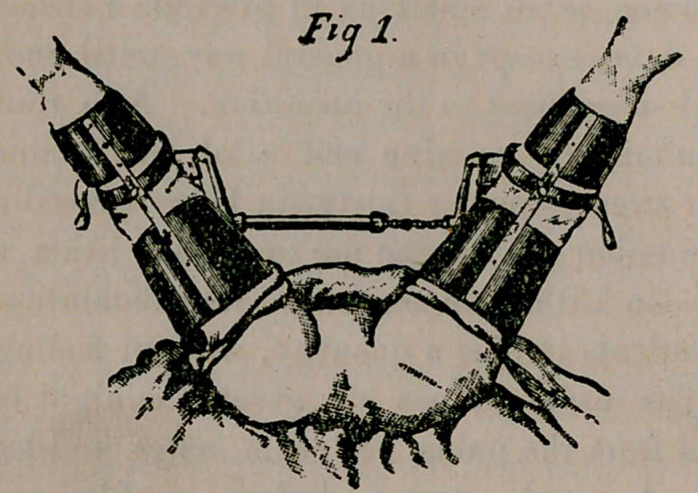


**Fig. 2. f2:**